# Naringin Alleviates Glucose-Induced Aging by Reducing Fat Accumulation and Promoting Autophagy in *Caenorhabditis elegans*

**DOI:** 10.3390/nu15040907

**Published:** 2023-02-10

**Authors:** Peisen Guo, Panpan Wang, Limin Liu, Peixi Wang, Guimiao Lin, Zhi Qu, Zengli Yu, Nan Liu

**Affiliations:** 1 College of Public Health, Zhengzhou University, Zhengzhou 540001, China; 2 Institute of Chronic Disease Risks Assessment, School of Nursing and Health, Henan University, Kaifeng 475004, China; 3 Institute of Environment and Health, South China Hospital, Medical School, Shenzhen University, Shenzhen 518116, China; 4 School of Public Health, Medical School, Shenzhen University, Shenzhen 518061, China

**Keywords:** naringin, glucose, anti-aging, fat accumulation, autophagy

## Abstract

Naringin (Nar) is a dihydroflavonoid compound, widely found in citrus fruit and used in Chinese herbal medicine. As a phytochemical, it acts as a dietary supplement that can delay aging and prevent aging-related disease, such as obesity and diabetes. However, its exact mechanism remains unclear. In this study, the high-glucose-induced (HGI) *Caenorhabditis elegans* model was used to evaluate the anti-aging and anti-obesity effects of Nar. The mean lifespan and fast movement span of HGI worms were extended roughly 24% and 11%, respectively, by Nar treatment. Oil red O staining revealed a significant reduction in fat accumulation and dFP::LGG-labeled worms showed the promotion of autophagy. Additionally, whole transcriptome sequencing and gene set variation analysis suggested that Nar upregulated the lipid biosynthesis and metabolism pathways, as well as the TGF-β, Wnt and longevity signaling pathways. Protein–protein interaction (PPI) network analysis identified hub genes in these pathways for further analysis. Mutant worms and RNA interference were used to study mechanisms; the suppression of *hlh-30*, *lgg-1*, *unc-51*, *pha-4*, *skn-1* and *yap-1* disabled the fat-lowering, lifespan-prolonging, and health-promoting properties of Nar. Collectively, our findings indicate that Nar plays an important role in alleviating HGI-aging and anti-obesity effects by reducing fat accumulation and promoting autophagy.

## 1. Introduction

Aging can be considered a progressive functional decline in tissues and organs, leading to a series of age-related diseases, such as neurodegenerative diseases, cardiovascular diseases, and cancer, with profound impacts on global human health [1,2,3]. The mechanisms of aging are multi-factorial. Some chronic metabolic diseases, such as obesity, can accelerate the progress of aging and shorten lifespan [4]. A cross-sectional study demonstrated that obesity accelerates brain aging in middle-aged humans [5]. Current studies on anti-aging are mainly based on cleaving the excess reactive oxygen species (ROS) in the human body. For example, many natural plant extracts can be employed as antioxidants to fight against aging and increasing evidence suggests that polyphenol phytochemicals have obvious anti-aging effects [6], such as delaying brain and skin aging [7,8]. 

Naringin (Nar) is a dihydroflavonoid polyphenol compound, widely found in Chinese herbal medicine and citrus fruit, with important medical and economic value due to its favorable effects of inhibiting inflammation and apoptosis to enhance the activity of endothelial cells [9] and as a potential therapeutic agent for obesity. Nar supplementation is beneficial to patients with dyslipidemia, controlling body weight, lipid profile and adiponectin levels [10]. However, the underlying mechanism concerning the anti-obesity effect is unclear.

The nematode, *Caenorhabditis elegans* (*C. elegans*), is a commonly used model organism with a small body size, a short lifespan, and a completely sequenced genome [11]. Previous studies have shown that high-glucose-induced (HGI) nematodes are an excellent model for exploring the mechanisms of aging [12] and obesity [11]. HGI nematodes have phenotypes corresponding with the aging process, such as lipofuscin accumulation and locomotion decline [13]. In addition, increasing evidence demonstrates that the life span of nematodes can be prolonged by reducing the accumulation of fat. Extensive studies have reported that phytochemicals can regulate fat metabolism. For example, curcumin can reduce fat accumulation by downregulating the genes of *sbp-1* and *fat-6* [14], and polysaccharides from volvacea can reduce fatty acid synthesis by inhibiting the *aak-2*/*nhr-49*-mediated pathway [15]. The genes, *fat-5*, *6* and *7*, as an ortholog of human SCD5, control the generation of unsaturated fatty acids (UFAs) in *C. elegans*. A decreased expression of these three genes reduces lipid droplet size [16].

Autophagy is an evolutionarily conserved pathway to degrade and recycle cellular components [17]. It is important to the development of *C. elegans* and an increase in autophagy can prolong lifespan. For example, the upregulation of autophagy by astaxanthin induction can extend the lifespan of *C. elegans* by promoting the expression of *hlh-30* or *daf-16* [18]. Cannabidiol can also induce longevity by regulating genes correlated with autophagy, such as *bec-1* and *unc-51* [19]. In addition, autophagy can modulate lipid metabolism to extend the lifespan [20]. The suppression of ATG-9A, a *C. elegans* ortholog of ATG-9 in humans, which is involved in phagophore formation, can inhibit autophagy and thereby increase the size and number of lipid droplets [17,21]. Furthermore, saponin can reduce fat accumulation through autophagy pathways, such as the *hlh-30* pathway, which ultimately improves health and extends lifespan [22]. A previous study has demonstrated that naringin can extend the lifespan of worms [23]. However, as a phytochemical, it is not clear whether Nar, like saponins, reduces fat deposition and promotes the autophagy pathway to alleviate glucose-induced aging and improve the corresponding phenotypes.

In this work, we explored the beneficial effects of Nar on HGI nematodes by testing aging-related changes, including lifespan, fast movement span, fat accumulation and autophagy. The underlying regulated mechanisms were also revealed, enabling us to better understand whether Nar has anti-aging and anti-obesity properties via autophagy to ameliorate glucose-induced life-shortening and metabolic disorders such as diabetes and obesity. Elucidating these mechanisms also provides a basis for promoting human health and longevity.

## 2. Materials and Methods

### 2.1. C. elegans Maintenance

All *C. elegans* strains were cultured following standard protocols on nematode growth medium (NGM) containing a food source of OP50 *Escherichia coli* and at 20 °C, unless otherwise indicated [24]. Synchronized young adults were acquired by a previously described method [25]. Briefly, worms in an egg-laying period were collected in a centrifuge tube using 3.5 mL of M9, then 1.5 mL of lysate (5 mL 5M NaOH + 10 mL 5% NaClO) was added and the mixture gently shaken for 2–3 min. Tubes were then centrifuged for 1 min at 3000 rpm; the procedure was repeated twice. After that, the mixture was washed three times with 5 mL of M9. The obtained eggs were shaken at 30 rpm overnight in M9 to incubate and obtain L1 nematodes. The L1 nematodes were seeded onto NGM medium, and the synchronized L4 nematodes could be obtained 48 h later. In addition, Nar was dissolved in DMSO and added to the NGM before pouring, producing a final concentration of DMSO that was controlled at 0.1%. To induce worm aging, 2% glucose was used [26]. *C. elegans* strains used in this study were the wild-type N2, Bristol (wild-type); DLM1 (dFP::LGG-1); JIN1375 (hlh-30(tm1978) IV).

### 2.2. Lifespan and Fast Movement Span Analysis

Fast movement span, which is indicative of a decline in motor ability with age, was estimated using previously published methods [27]. Worms with continuous, coordinated, forward sinusoidal movements when tapping the Petri dishes against the microscope stage were classified as having fast movement, with fast and non-fast movements considered as the outcome events. Moreover, nematodes that did not respond to platinum wire stimulation and drilled into the NGM or climbed the Petri dish wall were recorded as dead and censored, respectively. Synchronized L4 worms were transferred to NGM plates and seeded with OP50 *E. coli*. Nematodes were transferred to new NGM plates every 1–2 d and the non-fast movement, dead or censored ones were recorded at the same time. The log-rank test was applied to compare overall survival rates and fast movement rates among treatments. 

### 2.3. RNA Interference (RNAi)

RNAi was generated by feeding *C. elegans* with HT115 bacteria transformed with an L4440 vector for expression of the double-stranded RNA (dsRNA) of genes of interest. Clones used from the *C. elegans* RNAi feeding library [28] were *lgg-1* (C32D5.9), *unc-51* (Y60A3A.1), *skn-1* (T19E7.2), and *pha-4* (F38A6.1). For the gene *yap-1*, the whole coding sequence was cloned into the vector L4440 and expressed in HT115. All five RNAi clones were verified by sequencing. Clones were grown overnight at 37 °C in Luria broth with 100 µg/mL of ampicillin at 200 rpm. For RNAi feeding plates, NGM plates were prepared with the addition of 1 mM of isopropyl β-D-1-thiogalactopyranoside to induce dsRNA expression. To prevent the development of nematodes from being affected, RNAi feeding started at the L4 stage and then continued throughout the lifespan. The empty vector L4440 was used as the negative control for all RNAi experiments.

### 2.4. Analysis of Auto-Fluorescence of C. elegans

Synchronized L4 worms were transferred to NGM plates with or without treatments. On days 5 and 9, nematodes were transferred individually to slides prepared with 5 mM of levamisole hydrochloride. After being anesthetized and covered with cover slides, worms were photographed (200× magnification) under an inverted fluorescence microscope (Ti2, Nikon, Tokyo, Japan) with a Texas filter. Then, the red channel was split out and a uniform threshold was set to quantify the red fluorescence intensity by ImageJ (NIH) software.

### 2.5. Fat Accumulation Testing with Oil Red O Staining

To measure fat accumulation, we used oil red O staining, using a slightly modified protocol from that previously used [29]. Synchronized L4 worms were cultured on NGM plates with different treatments for 5 d at 20 °C. Worms were then collected and washed in M9 with 0.05% Triton X-100 three times. The worms were centrifuged at 560× *g* for 1 min and the supernatant was removed, leaving approximately 100 µL. Then, 600 µL of 40% isopropanol was added to the worm pellet and rocked at room temperature for 3 min. The above centrifugation steps were repeated. Subsequently, the worms were stained with 600 μL of Oil Red O working solution (Beyotime, Shanghai, China) for 2 h at room temperature at 30 rpm. After washing, worms were imaged by a stereomicroscope (55 × magnification) outfitted with a color camera (S9i, Leica, Wetzlar, Germany). The color picture was converted to an 8-bit file and the whole worm was carefully selected using the polygon selections tool using ImageJ, and its optical density was then quantified.

### 2.6. Physiological Phenotype Analysis of C. elegans

Nematodes synchronized to the L4 stage were transferred to NGM plates with various treatments, and their body length and width were measured on days 2, 5 and 9 under an inverted fluorescent microscope (Ti2, Nikon, Tokyo, Japan) (100× magnification). Body length and width were quantified by ImageJ. 

For brood number analysis, approximately 30 nematodes in the L4 stage were transferred to each treatment group, placing a single nematode per NGM medium. The nematodes were transferred to the new NGM at a fixed time every day until no more eggs were laid. The media with eggs were placed at 20 °C, and counted using a stereomicroscope (S9i, Leica, Wetzlar, Germany) (20× magnification) after 3 d.

The frequency of body bends and head swings were measured 2, 5 and 9 d after treatment began using a stereomicroscope (S9i, Leica, Wetzlar, Germany) (20× magnification). Individual nematodes were transferred to M9 drops one at a time. The head and tail swung to the left and right at the same time once for counting. Then, the nematode was transferred to NGM medium without OP50 *E. coli*, and its body bend frequency was observed after the nematode had settled for 1 min. During movement, a sine wave formed along the nematode’s body axis in the movement direction was counted as one body bend. 

The frequency of pharyngeal pumping was measured using a previously published protocol [30]. Briefly, the nematode was individually picked onto the middle of a fresh OP50-seeded NGM and the grinder movement was counted manually over 20 s under an optical microscope (RTX, Rixin Optics Co., Shangrao, China) (100× magnification) 2, 5 and 9 d after treatment began. 

### 2.7. Rhodamine 6G Staining

H_2_O_2_ produced by lipid β-oxidation can lead to the generation of ROS, the accumulation of which impairs mitochondrial function, such as the decline of mitochondrial membrane potential (MtMP), and affects the lifespan of nematodes, as well as contributing to type 2 diabetes and obesity in humans [31,32]. MtMP was measured to reveal changes in mitochondrial activity using a previously published protocol [33]. Briefly, synchronized L4 worms were cultured on the NGM plates of each treatment group for 5 d. Next, the nematodes were transferred to a plate with NGM and Rhodamine 6G for 4 h. Nematodes were then placed onto a glass slide with an agar pad and immobilized by 5 mM levamisole hydrochloride. Finally, they were observed and photographed (100× magnification) under an inverted fluorescence microscope (Ti2, Nikon, Tokyo, Japan) with Texas filter. Then, the red channel was split out and a uniform threshold was set to quantify the red intensity using ImageJ software.

### 2.8. Observing Autophagy by dFP::LGG

Synchronized transgenic nematodes, dFP::LGG, were treated with or without Nar under a high-glucose condition for 5 d. Then, 4% paraformaldehyde was used to immobilize nematodes with a coverslip. Images were captured using an inverted fluorescence microscope (Ti2, Nikon, Tokyo, Japan) with an FITC filter (100× magnification). Then, the green channel was split out and a uniform threshold was set to quantify the green fluorescence intensity in ImageJ.

### 2.9. Whole Transcriptome Sequencing Analysis

Synchronized L4 worms were cultured on NGM with the various treatments, incubated at 20 °C for 5 d, and then transferred to new NGM plates for each treatment every day using M9 and natural sedimentation to remove progeny. Approximately 1–2 μg total RNA of *C. elegans* was extracted in each sample. The integrity of the total RNA was confirmed by agarose gel electrophoresis, and then quantification and quality control were performed using NanoDrop^®^ (ND-1000, Thermo-Fisher, Waltham, USA). RNA was first enriched by NEB Next^®^ Poly(A) mRNA Magnetic Isolation Module (New England Biolabs, Ipswich, USA). Next, the library was constructed using the KAPA-stranded RNA-Seq library prep kit (Illumina, Santiago, USA) and controlled with Bioanalyzer (G2938C, Agilent, Santa Clara, USA). The library was degraded to single-strand DNA (ssDNA) by 0.1 M NaOH and diluted to 8 pM. Subsequently, the in situ amplification of ssDNA was carried out with the NovaSeq 6000 S4 reagent kit (300 cycles) (15057934, Illumina, Santiago, USA). The product was sequenced by Illumina NovaSeq 6000 and the trimmed data were aligned to the reference genome (WBcel235) by Hisat2 (2.1.0) [34]. The StringTie (1.3.3) software [35] was applied to estimate transcription abundance and obtain the count data of genes and transcripts. Differentially expression analysis was conducted with the R package “Ballgown” (2.10.0) [36,37]. The mRNAs with a mean expression in all samples of greater than 1 were kept for further analysis. CircRNAs were identified and quantitated using the software STAR (2.5.2b) [38] and CIRCexplorer2 (2.3.2) [39]. Count data of circRNA were used for the differentially expressed analysis using the “edgeR” R package. 

The miRNA library of *C. elegans* was constructed using the Multiplex Small RNA Library Prep Set for Illumina (E7508L, New England Biolabs, Ipswich, USA). After degrading miRNA to ssDNA, ssDNA was captured in an Illumina flow cell for in situ amplification. It was sequenced on the Illumina NextSeq 500 platform (50 cycles). Other steps were similar to that for the mRNA sequencing above. After quality control by Solexa CHASTITY and discarding the 3′ adapter and short fragments (<17 nt) in reads by Cutadapt (1.14), mirdeep2 (0.0.8) was used to compare the trimmed miRNA data with the reference genome and to quantify its expression. The count per million value of miRNA was applied to conduct differentially expressed analysis using the “edgeR” R package.

The screening of differentially expressed mRNA (DEmRNA) used the standards of |log2FC| > 0.138 and *p* < 0.05, while for the differentially expressed lncRNA (DElncRNA), circRNA (DEcircRNA), and miRNA (DEmiRNA), standards used were |log2FC| > 0.585 and *p* < 0.05.

### 2.10. qPCR Analysis

Synchronized L4 worms were cultured on plates at 20 °C for 5 d and then transferred to new treatment NGM plates each day using M9 and natural sedimentation to remove progeny. Then, worms were collected, and the total RNA of worms (~1000 worms per sample) was extracted using a kit (RC101-01, Vazyme, Nanjing, China) and converted to Cdna using HiScript III RT SuperMix for Qpcr (+gDNA wiper) (R323-01, Vazyme, Nanjing, China). The cDNA was diluted 20 times for qPCR. Primers were designed using the NCBI database and are listed in Table S1. qPCR was conducted using AceQ Universal SYBR qPCR Master Mix (Q511, Vazyme, Nanjing, China) and QIAquant96 5plex (QIAGEN, Hilden, Germany). The relative expression levels of the genes were assessed using the 2^−ΔΔCT^ method and normalized to the expression of *act-1* [40].

### 2.11. Statistical Analysis

Two-tailed t-tests (where data were normally distributed), Welch t test (where data were normally distributed but not variance homogeneity), and Mann–Whitney were used to test for differences between two groups and calculate *p*-values. Kaplan–Meier and log-rank tests were used to display and analyze the survival curve. All statistical analyses and data visualization were carried out using R software (4.1.2).

## 3. Results

### 3.1. Anti-Aging Effect of Nar on HGI Worms

**Figure 1:**
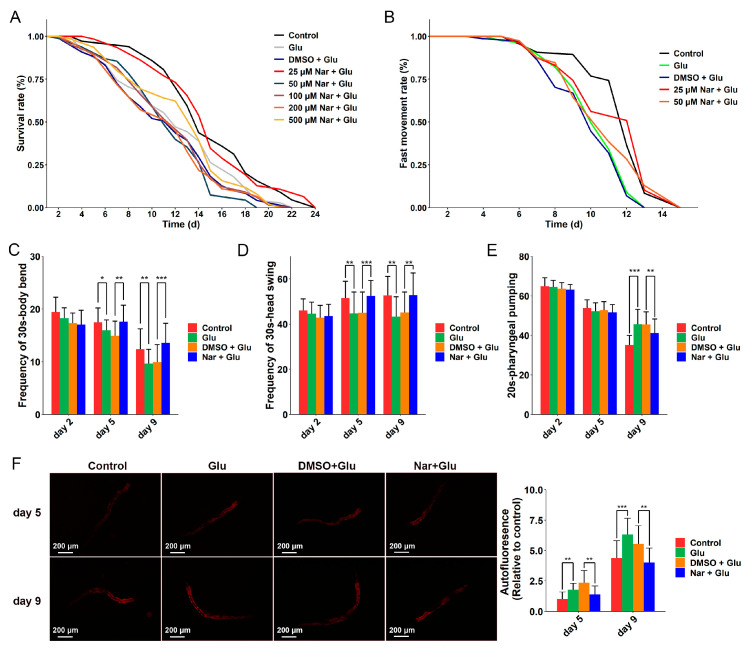
Pathophysiological analysis of *C. elegans* in four different treatments: control, glucose (Glu) induction, DMSO + Glu and Nar + Glu, showing that Nar relieved Glu-induced accelerated aging. (**A**) Survival curves of the four different treatment groups. (**B**) The fast movement curves of different treatment groups. (**C**) The frequency of body bend (30s); *n* ≥ 27 worms per group for day 2, *n* ≥ 21 for day 5, and *n* ≥ 25 for day 9. (**D**) Frequency of head swings (30s); *n* ≥ 29 worms per group for day 2, *n* ≥ 21 for day 5, and *n* ≥ 24 for day 9. (**E**) Frequency of pharyngeal pumps (20s); *n* ≥ 32 worms per group for day 2, 5 and 9. (**F**) Auto-fluorescence of nematodes in different treatment groups on days 5 and 9; *n* ≥ 12 worms per group for day 5, and *n* ≥ 17 for day 9. Data are representative one of two independent experiments and represented mean ± SD. The symbols *, ** and *** represent *p* < 0.05, 0.01, and 0.001, respectively.

We first investigated the beneficial effects of Nar on HGI *C. elegans*. Gradient concentrations of 25, 50, 100, 200 and 500 μM Nar were tested to find the optimum. The addition of 25 μM Nar prolonged the mean lifespan of *C. elegans* most strongly (Figure 1A), increasing mean lifespan by approximately 24% compared to the DMSO group (Table 1). This dose–response effect is consistent with tomatidine [41] and shatavarin IV [42], whereby the lifespan-prolonging effect disappeared at higher concentrations. Moreover, 50 μM Nar extends the lifespan of worms under normal conditions [23], indicating that a lower dose of Nar could prolong the lifespan under a high-glucose condition, which suggests that there are other mechanisms that remain to be explored. The 25 μM Nar also improved aging-related phenotypes. The mean fast movement span was extended by approximately 11%, (Figure 1B and Table 2) suggesting that HGI worms tended to be healthier. Thus, 25 μM Nar was used for all subsequent experiments. The frequency of body bends and head swings in HGI worms with Nar was significantly increased on days 5 and 9 compared to the control (Figure 1C,D). We then explored whether longevity is mediated by dietary restriction-like effects by recording the frequency of the pharyngeal pump. No significant difference in pharyngeal pump frequency was seen in Nar-fed nematodes at days 2 and 5, but at 9 d, pharyngeal pump frequency significantly decreased in Nar-fed nematodes (Figure 1E), indicating that Nar may not prolong lifespan through dietary restriction-like effects during the early stage of the nematode lifespan. Moreover, autofluorescence in HGI worms fed with Nar was significantly lower compared to those without Nar on days 5 and 9 (Figure 1F). These results suggest Nar has anti-aging effects on HGI worms.

### 3.2. Analysis of Transcript Sequencing

**Figure 2:**
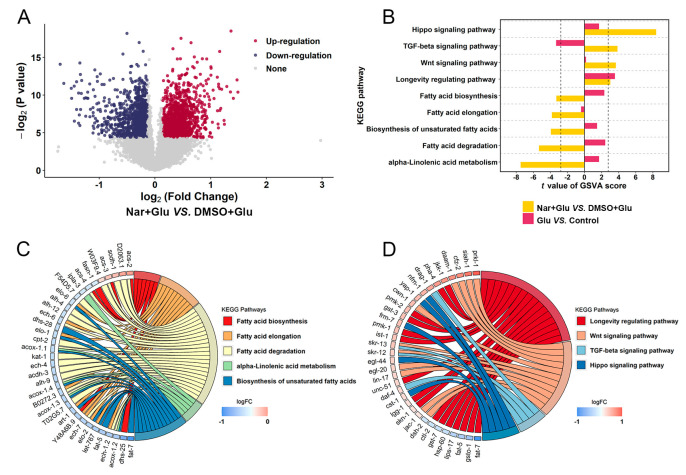
Bioinformatic analysis for whole transcriptome sequencing. (**A**) Volcano plot showing differentially expressed genes between “DMSO + Glu” and “Nar + Glu” groups. (**B**) Nine significantly different pathways screened by GSVA. (**C**) The specific genes in upregulated and (**D**) downregulated pathways from the nine pathways in (**B**).

**Figure 3:**
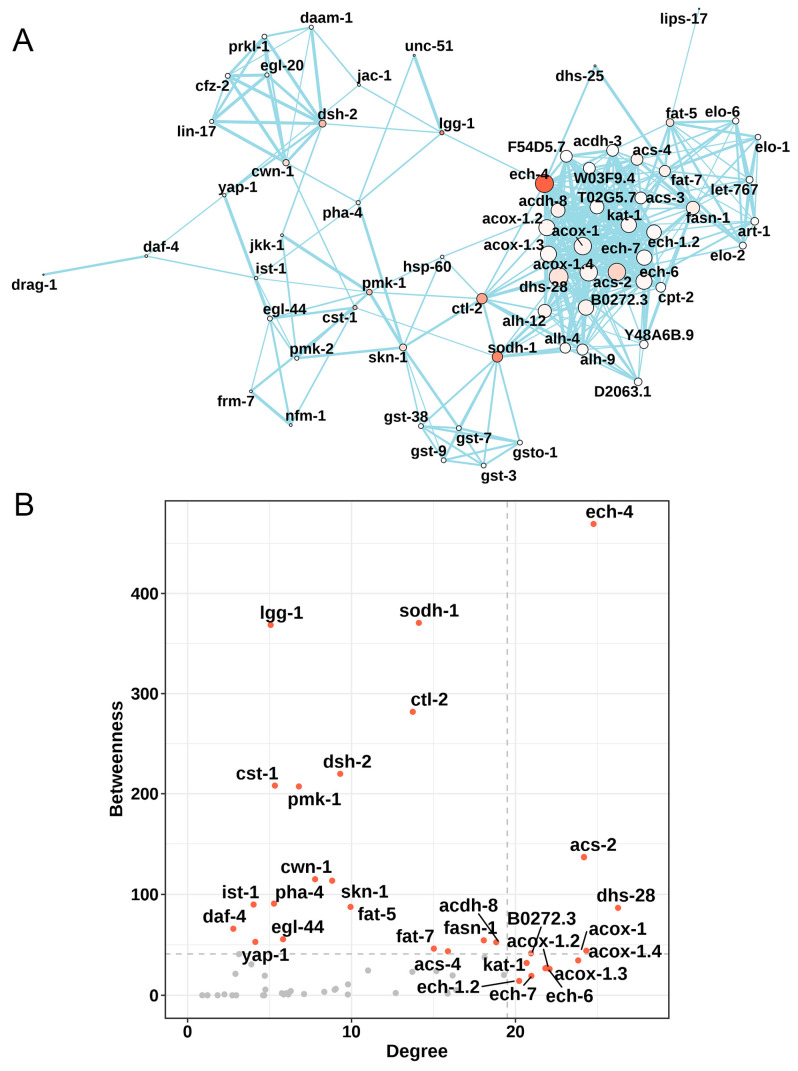
PPI network for identifying hub genes. (**A**) Construction of the PPI network was based on the differentially expressed genes in nine signaling pathways identified in Figure 2C,D. The network was constructed by STRING (https://cn.string-db.org/ (accessed on 5 September 2022)) and visualized by R software. (**B**) Hub genes in the PPI network were identified based on the values of betweenness centrality and degree centrality of a node in the network, where a gene represented a node.

A total of 2603 mRNAs (Figure 2A), 4 lncRNAs (Figure S1A), 2 circRNAs (Figure S1B), and 32 miRNAs (Figure S1C) were differentially expressed between the HGI worms with and without 25μM Nar. Based on 126 KEGG pathways, the gene set variation analysis (GSVA) algorithm revealed 48 notable changes in the pathway expression levels when HGI worms were treated with Nar (Figure S2)—the *t*-value and the specific genes in these 48 pathways are listed in Table S2. Then, nine signaling pathways related to the study purpose were screened (Figure 2B). For example, fat metabolism-related pathways were downregulated, while Wnt and TGF-β, as well as longevity signaling pathways, were upregulated. The differentially expressed genes in these pathways are displayed in Figure 2C,D and Table S3. To identify appropriate genes for subsequent analysis, a protein–protein interaction (PPI) network analysis was performed on these genes (Figure 3A). Key genes with betweenness > 41 and degree > 19.5 were selected (Figure 3B).

### 3.3. Improvement of Reproduction and Development in HGI C. elegans

**Figure 4:**
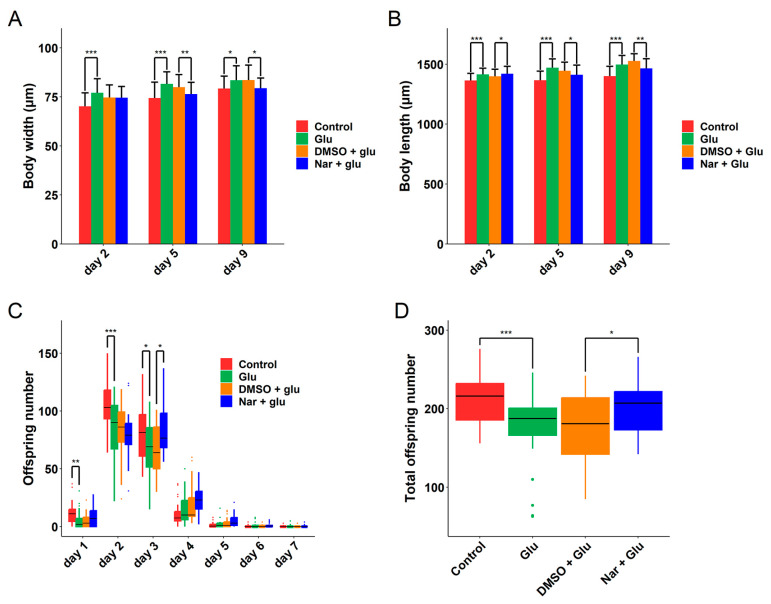
Nar improved the development and reproduction of HGI worms. (**A**) The body width and (**B**) length of different treatment groups on days 2, 5 and 9; *n* ≥ 66 worms per group for day 2, *n* ≥ 43 for day 5, and *n* ≥ 21 for day 9. (**C**) The daily and (**D**) total number of offspring during the reproductive period of each group (*n* ≥ 29 worms per group). All experiments were replicated twice. Data are representative one of two independent experiments and represented mean ± SD. The symbols *, ** and *** represent *p* < 0.05, 0.01, and 0.001, respectively.

Previous studies have demonstrated that the high concentration of glucose (50 mM) in *C. elegans* can reduce body size and brood size of adult worms [43]. Our transcript analysis suggests that the reproduction and development of HGI *C. elegans* can be improved by Nar (Figure 2B). We found that the body length and width of HGI worms were significantly higher than the normal control on days 2, 5, and 9 (Figure 4A,B). Interestingly, although the body length of worms treated with Nar were longer than that of the HGI control worms on day 2, they were shorter on days 5 and 9, suggesting that the HGI worms grow faster than those with Nar treatment. Nar did reduce the decline in offspring number of the HGI worms, especially on the 3rd day (Figure 4C,D). 

### 3.4. Reduction in Fat Accumulation in HGI Worms

**Figure 5:**
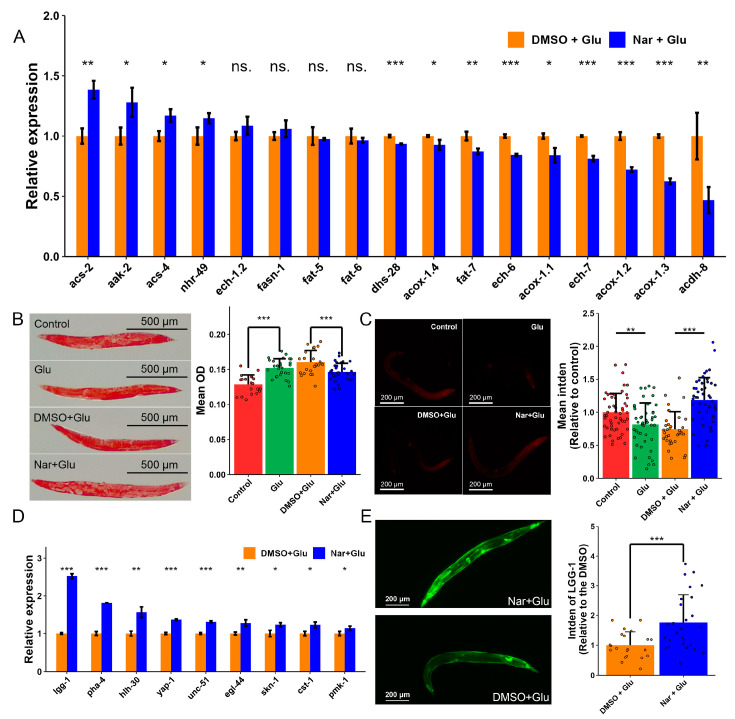
Fat accumulation and autophagy in HGI worms treated with Nar. (**A**) qPCR analysis confirms the difference in lipid-related gene expression of HGI worms with and without Nar administration (*n* = 3 samples per group). The experiment was replicated three times. (**B**) Observations of fat accumulation, and its reduction by Nar, in HGI *C. elegans* model by Oil red O staining (*n* ≥ 20 worms per group). The experiment was replicated three times. (**C**) Observations of reduced mitochondrial dysfunction with Nar in HGI *C. elegans* model by R6G staining (*n* ≥ 35 worms per group). The experiment was replicated two times. (**D**) qPCR confirmation of differentially expressed longevity-related genes in HGI worms with or without Nar administration (*n* = 3 samples per group). The experiment was replicated three times. (**E**) Fluorescence quantification and comparison of the dFP::LGG worms with or without Nar treatment (*n* ≥ 20 worms per group). The experiment was replicated two times. Data are representative one of at least two independent experiments and represented mean ± SD. The symbols ns., *, ** and *** represent no significance, *p* < 0.05, 0.01, and 0.001, respectively.

Sequencing analysis revealed that Nar might be involved in fat biosynthesis and metabolism (Figure 2B). Thus, we performed qPCR to confirm the expression level of several genes associated with fat regulation (Figure 5A). Four genes, *acs-2*, *aak-2*, *acs-4*, and *nhr-49*, were upregulated and nine genes, including *fat-7* and *ech-6*, were downregulated. The fact that fat accumulation in HGI worms is attenuated by Nar treatment was confirmed using oil red O staining (Figure 5B). Moreover, a large amount of ROS would be produced during fat metabolism, such as β-oxidation, which damages mitochondrial function and thereby shortens the lifespan of nematodes. Fat accumulation undoubtedly contributes to ROS production and Nar can cleave excessive ROS [23]. We thus tested MtMP to reveal the mitochondrial activity by Rhodamine 6G staining, which indicated that Nar significantly ameliorated mitochondrial damage induced by glucose (Figure 5C).

### 3.5. Induced Autophagy against High Concentration of Glucose

The bioinformatic analysis confirmed that the longevity pathway in *C. elegans* was upregulated by Nar. We performed qPCR analysis on several key genes involved in lifespan regulation and autophagy induction (Figure 5D). The expression of autophagy-related genes including *hlh-30*, *lgg-1*, *unc-51* and *pha-4* was significantly increased in the Nar treatment. The dFP-labeled worms further demonstrated that Nar could induce autophagy (Figure 5E).

### 3.6. Reduced Fat Accumulation by Nar Might Be through Autophagy Pathway

**Figure 6:**
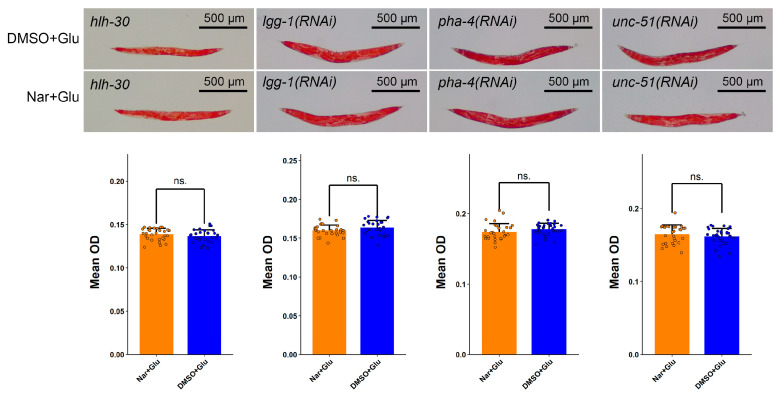
The fat-lowering effect of Nar was absent when autophagy-related genes were suppressed using mutants or RNAi worms, including *hlh-30* mutants, *lgg-1* RNAi, *pha-4* RNAi and *unc-51* RNAi (*n* ≥ 29 worms per group). The experiment was replicated two times. Data are representative of one of two independent experiments and represented mean ± SD. The symbols ns. represent no significance.

**Figure 7:**
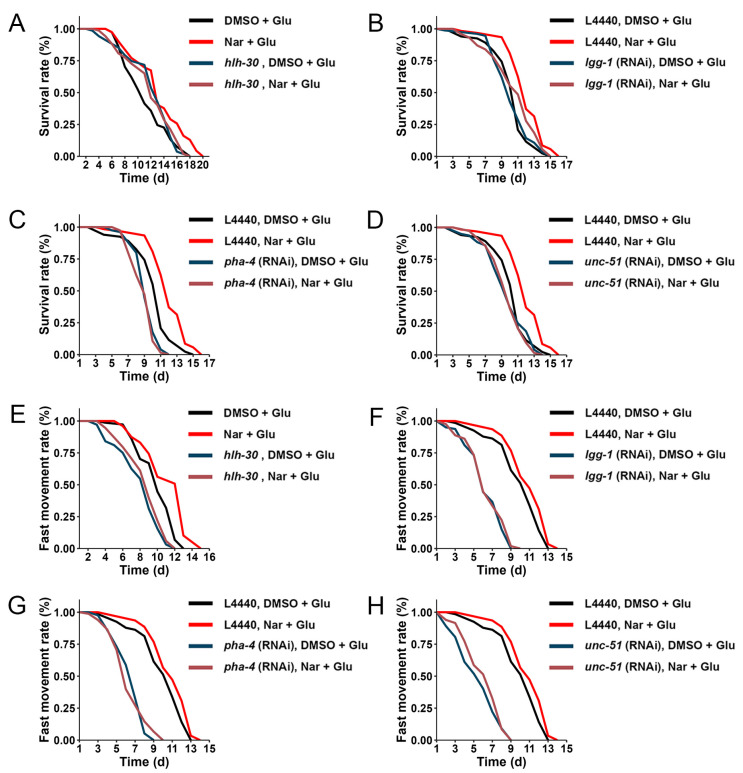
The survival and fast movement rate of *hlh-30* mutants and *C. elegans* fed with RNAi bacteria, *lgg-1*, *pha-4* and *unc-51*, in different treatments. HGI worms with or without Nar treatment did not differ in survival rate when using (**A**) *hlh-30* mutants or when fed RNAi bacteria including (**B**) *lgg-1*, (**C**) *pha-4* and (**D**) *unc-51*. Similarly, HGI worms with or without Nar treatment did not differ in fast movement rate when using (**E**) *hlh-30* mutants or when fed RNAi bacteria including (**F**) *lgg-1*, (**G**) *pha-4* and (**H**) *unc-51*. Experiments were replicated three times. Data are representative one of three independent experiments.

Previous studies have shown that autophagy can be involved in regulating lipid metabolism. Combined with the related clues given above, we speculated that Nar could reduce fat accumulation by inducing autophagy. An *hlh-30* mutant and three other autophagy-related RNAi treatments were employed to confirm our hypothesis. The fat-lowering effect of Nar was absent in *hlh-30* mutants and in *lgg-1*, *unc-51* and *pha-4* RNAi-exposed worms (Figure 6), concomitant with the elimination of the life- and health-prolonging effects of Nar (Figure 7A–H, Table 3 and Table 4). This implies that the amelioration of HGI senescence by Nar is due to the reduction in fat accumulation through the promotion of autophagy.

### 3.7. skn-1 and yap-1 Genes Mediating Fat Reduction Might Be Involved in Delaying Glucose-Induced Aging

**Figure 8:**
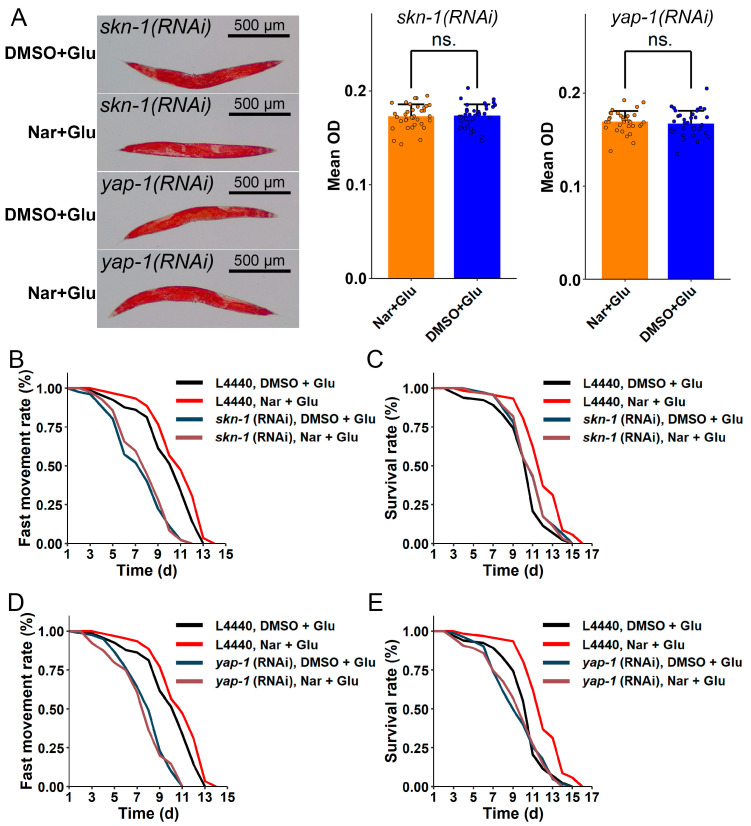
Effects of Nar on HGI worms mediated by *skn-1* and *yap-1.* (**A**) Fat-lowering effect of Nar was blocked by *skn-1* and *yap-1* RNAi treatment in HGI worms, as observed by oil red O staining (*n* ≥ 33 worms per group). The experiment was replicated two times. The data are represented mean ± SD. No differences were found between HGI worms with or without Nar treatment in fast movement rates or survival rates for nematodes treated with *skn-1* RNAi (**B**,**C**) and *yap-1* RNAi (**D**,**E**). The experiment was replicated three times. Data are representative one of at least two independent experiments. The symbols ns. represent no significance.

The longevity and Hippo signaling pathway genes, *skn-1* and *yap-1*, were up-regulated in HGI worms treated with Nar (Figure 2D and Figure 5D). Thus, we hypothesized that fat storage could be decreased by Nar through modulating *skn-1* and *yap-1*. To test this hypothesis, we knocked down these two genes and, as predicted, the fat-lowering property of Nar disappeared (Figure 8A). Moreover, the fast movement span and lifespan no longer differed significantly between HGI worms with or without Nar treatment (Figure 8B–E, Table 3 and Table 4). These results reveal that the SKN-1 and YAP-1 pathways are involved in the anti-obesity and anti-aging properties of Nar.

## 4. Discussion

**Figure 9:**
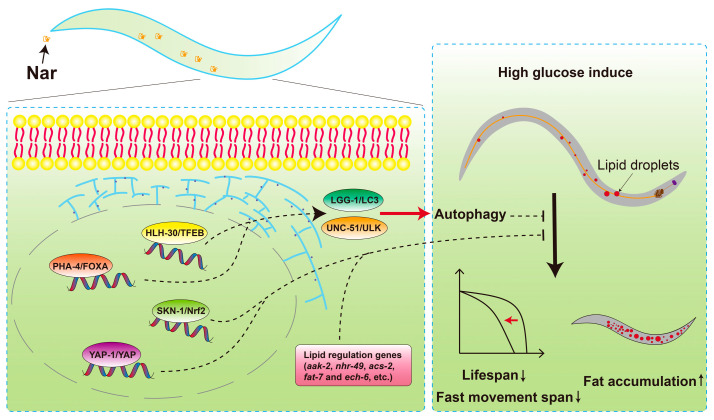
A graphical abstract of the molecular mechanisms and functions underlying the effects of Nar on *C. elegans* to promote autophagy and reduce fat accumulation.

Nar has well-known health properties, including anti-inflammatory, anti-aging, and anti-oxidant properties. However, the specific mechanisms responsible remain unclear. In this study, we established the HGI *C. elegans* model to assess changes in aging-related phenotypes following Nar administration. Using whole transcriptome sequencing, we show significantly changed genes associated with lipid regulation and autophagy, such as in the genes *skn-1*, *hlh-30*, *lgg-1*, *unc-51* and *pha-4*. Knocking out these genes led to the disappearance of fat-lowering, health-promoting and life-prolonging effects from Nar. Collectively, our findings demonstrate that Nar produces anti-obesity and anti-aging functions in HGI worms through autophagy inductions (Figure 9).

The present study revealed that Nar reduces fat accumulation in HGI worms by promoting autophagy. Some genes involved in autophagy processing were significantly upregulated, including *hlh-30*, *lgg-1*, *unc-51*, and *pha-4.* HLH-30, a *C. elegans* orthologue of TFEB, is a conserved transcript regulator controlling multiple genes associated with the autophagy process in *C. elegans* [44]. Many bioactive substances extend the lifespan of *C. elegans* by stimulating *hlh-30* to induce autophagy [18,22]; for example, the human LC3 ortholog gene, *lgg-1*, is a key downstream gene of *hlh-30* that is crucial to the formation of the autophagosome [17]. Both *unc-51* (a *C. elegans* orthologue of ULK1) and *lgg-1* are involved in lipid regulation [45]. Phytochemicals could enhance autophagy-related genes and regulate fat storage and consumption. For example, shatavarin IV is a powerful therapeutic agent that can reduce fat accumulation and promote dietary restriction-induced autophagy mediated by *pha-4*, a transcript factor and *C. elegans* orthologue of FOXA [46]. Resveratrol also possesses both autophagy-inducing and lipid-reducing activities [47,48]. Bai has established a linkage between lipid regulation and autophagy, revealing that saponins from bitter melon exert fat-lowering effects by promoting the *hlh-30*-mediated autophagy process [22]. Likewise, we integrated autophagy induction with lipid reduction caused by Nar, indicating that autophagy-related genes, including *hlh-30*, *lgg-1*, *unc-51*, and *pha-4*, are involved in anti-obesity and anti-aging effects.

RNA-sequencing and qPCR confirmed the related genes of *skn-1*, *yap-1,* and *egl-44*. The *C. elegans* orthologues of Nrf2 and YAP1 were up-regulated in HGI worms with Nar treatment. It has been reported that excessive fat accumulation caused by abnormal conditions can activate the SKN-1/Nrf signaling pathway in *C. elegans* [49,50]. *Skn-1* is involved in the regulation of fat reduction caused by biologically active substances [51]. Thus, our results agree with the previous studies. In addition, YAP-1 is part of a highly conserved Hippo signaling pathway that combines with EGL-44 to function [52]. Saul [53] observed that the down-regulation of *yap-1* could shorten the mean lifespan of worms. However, the relationship between *yap-1* and fat accumulation remained unclear. Thus, we tested whether Nar could affect lipid metabolism to prolong the lifespan of HGI worms through the YAP-1 pathway. With the deletion of *yap-1* expression, Nar treatment no longer significantly decreased the fat mass of HGI worms. In addition, Nar did not affect the fast movement and life span of HGI worms with *yap-1* RNAi.

Our results reveal that several genes in pathways related to lipid regulation were upregulated with Nar administration, including genes *aak-2*, *nhr-49*, and *acs-2*, the *C. elegans* orthologue of human AMPK, PPAR-α, and ACSF2, respectively. Our result thus agrees with previous research that has suggested that the activation of *aak-2* can stimulate the expression of *nhr-49* to upregulate *acs-2* and thereby enhance fat consumption and reduce fat storage [54,55]. The *fat-7* encoding a stearoyl-CoA desaturase, also known as Δ9 desaturase, introduces a double bond into the saturated fatty acyl chain to control the biosynthesis of UFAs, which also participates in the enlargement of lipid droplets in *C. elegans* [16,56]. In contrast to our results, which showed that Nar led to the upregulation of *nhr-49* but the downregulation of *fat-7*, previous studies have found that *nhr-49* can stimulate *fat-7* to inhibit lipid metabolism, suggesting that the fat-lowering effect of Nar is partially dependent on the *aak-2*/*nhr-49*-mediated pathway. Nonetheless, evidence increasingly indicates that the downregulation of both *fat-7* and *ech-6* (an ortholog of human ECHS1) plays a role in the lipid-reducing effects of Nar [57,58].

GSVA showed that the TGF-β and Wnt signaling pathway are also regulated by Nar. These two pathways govern the development and reproduction of *C. elegans*. The TGF-β pathway plays a leading role in regulating *C. elegans*’ body length and width [59]. Previous studies have shown that the body length and width of HGI worms are smaller than that of control worms as larvae [43]. However, the regulation of TGF-β by Nar might allow for the HGI worm to recover its body length to some extent, resulting in the body length of the Nar treatment group on day 2 being longer than that of the HGI treatment group. However, by days 5 and 9, the control HGI worms had outgrown the Nar-treated worms in body length. Studies have shown that the body length of nematodes increases gradually from days 3 through to 14 [13]. Therefore, we can cautiously conclude that HGI accelerates aging in nematodes, while Nar can partially mitigate this effect. 

There are other mechanisms that cause a lower dose of Nar can prolong the lifespan of HGI nematodes. As we know, Nar affects the activity of several enzymes of carbohydrate metabolism, such as hexokinase, glucose-6-phosphatase, fructose-1,6-bisphosphatase, glycogen synthase, and glycogen phosphorylase [60]. Additionally, the results of GSVA (Table S2) also revealed that the signaling pathways of glycolysis/gluconeogenesis and citrate cycles (TCA cycle) in worms with Nar treatment are significantly lower than HGI worms at the pathway level. Thus, we speculated that Nar might inhibit the enzymes involved in glucose metabolism to interfere with the toxic effects of glucose. This mechanism, however, as the concentration of Nar increases, will lead to an increase in free glucose, enhancing the nonenzymatic reactions of glucose with proteins and nucleic acids to form advanced glycation-end products, ultimately shortening the lifespan of the nematode [61].

Worldwide, obesity has become a serious public health problem in humans. It is a multifactorial metabolic disorder and a risk factor for many health-related diseases, such as hypertension, diabetes, heart damage, cancer, cognitive decline, and COVID-19 complications [62,63,64,65]. Extensive studies also demonstrate that obesity holistically accelerates the aging process due to metabolic dysfunction [4,66]. Previous studies in the mouse model have shown that autophagy can promote the removal of lipid droplets, increase insulin secretion, and enhance insulin sensitivity, which can prevent diseases related to metabolic dysregulation [67]. Our study indicates that Nar can promote autophagy and reduce excessive fat accumulation by regulating *hlh-30*, *lgg-1*, *unc-51*, *pha-4*, *skn-1*, *yap-1*, and other genes associated with lipid levels, providing a theoretical basis for the prevention of diabetes and obesity. This study does have some potential limitations; in particular, the anti-aging and anti-obesity effects of Nar were estimated only in the model *C. elegans*. Nevertheless, all the genes that we have discussed have ortholog counterparts in mammals. Collectively, our study supports the potential value of Nar to promote human health as a nutritional supplement.

## 5. Conclusions

The present study indicates that Nar can improve health, prolong lifespan, and reduce the excessive fat accumulation of HGI *C. elegans* by stimulating genes associated with autophagy and fat regulation. These findings imply that Nar has the potential competence to ameliorate the effects of aging and obesity and improve the health of humans when used as a nutritional supplement.

## Figures and Tables

**Table 1:** Lifespan of *C. elegans* (N2) with different treatments.

Group	N	Events	Mean ± SE	Median	*p*-Value
Control	70	53	14.95 ± 0.57	14	-
Glu	81	74	12.09 ± 0.57	12	0.005
DMSO + Glu	92	74	11.29 ± 0.54	12	-
25 μM Nar + Glu	64	51	14.89 ± 0.60	15	<0.001
50 μM Nar + Glu	81	69	11.42 ± 0.45	11	0.632
100 μM Nar + Glu	83	69	11.87 ± 0.51	12	0.650
200 μM Nar + Glu	65	52	11.38 ± 0.60	12	0.921
500 μM Nar + Glu	68	54	12.73 ± 0.58	14	0.186

*Abbr:* Glu: Glucose; SE: Standard error.

**Table 2:** Fast movement span of *C. elegans* (N2) with different treatments.

Group	N	Events	Mean ± SE	Median	*p*-Value
Control	90	72	11.67 ± 0.22	12	-
Glu	81	62	10.28 ± 0.23	10	<0.001
DMSO + Glu	74	54	10.01 ± 0.25	10	-
25 μM + Glu	77	44	11.25 ± 0.32	13	0.001
50 μM + Glu	78	55	10.72 ± 0.30	11	0.060

SE: Standard error.

**Table 3:** Lifespan of HGI *hlh-30* mutants and various RNAi worms with or without Nar treatment.

Strain	Group	N	Events	Mean ± SE	Median	*p*-Value
*hlh-30*	DMSO + Glu	70	59	12.05 ± 0.46	13	
	Nar + Glu	73	56	11.95 ± 0.47	12	0.940
*lgg-1* *(RNAi)*	DMSO + Glu	80	62	10.21 ± 0.29	10	
	Nar + Glu	82	59	10.61 ± 0.36	11	0.146
L4440	DMSO + Glu	67	56	10.25 ± 0.31	11	
	Nar + Glu	62	47	12.07 ± 0.29	12	<0.001
*pha-4* (RNAi)	DMSO + Glu	81	72	9.36 ± 0.15	9	
	Nar + Glu	82	70	9.17 ± 0.14	9	0.328
*skn-1* (RNAi)	DMSO + Glu	82	65	10.97 ± 0.26	11	
	Nar + Glu	86	63	10.93 ± 0.25	11	0.703
*unc-51* (RNAi)	DMSO + Glu	83	68	9.68 ± 0.29	10	
	Nar + Glu	84	64	9.73 ± 0.26	10	0.640
*yap-1* (RNAi)	DMSO + Glu	80	64	9.53 ± 0.33	9	
	Nar + Glu	64	57	9.49 ± 0.37	10	0.979

*Abbr*: Glu: Glucose; SE: Standard error.

**Table 4:** Fast movement span of HGI *hlh-30* mutants and various RNAi worms with or without Nar treatment.

Strain	Group	N	Events	Mean ± SE	Median	*p*-Value
*hlh-30*	DMSO + Glu	70	65	8.05 ± 0.30	9	
	Nar + Glu	73	59	8.61 ± 0.27	9	0.214
*lgg-1* (RNAi)	DMSO + Glu	80	67	6.39 ± 0.22	6	
	Nar + Glu	82	64	6.48 ± 0.23	6	0.599
L4440	DMSO + Glu	67	56	10.03 ± 0.31	11	
	Nar + Glu	62	47	10.89 ± 0.28	11	0.034
*pha-4* (RNAi)	DMSO + Glu	81	77	6.53 ± 0.17	7	
	Nar + Glu	82	74	6.51 ± 0.21	6	0.353
*skn-1* (RNAi)	DMSO + Glu	82	68	7.48 ± 0.28	8	
	Nar + Glu	86	64	7.85 ± 0.26	8	0.522
*unc-51* (RNAi)	DMSO + Glu	83	75	5.54 ± 0.24	6	
	Nar + Glu	83	65	6.14 ± 0.23	7	0.141
*yap-1* (RNAi)	DMSO + Glu	80	64	7.99 ± 0.24	8	
	Nar + Glu	64	58	7.65 ± 0.29	8	0.653

*Abbr*: Glu: Glucose; SE: Standard error.

## Data Availability

RNA-seq and miRNA-seq data have been submitted to the Gene Expression Omnibus (GEO accession numbers GSE222361).

## Supplementary Materials

The following supporting information can be downloaded at: https://www.mdpi.com/article/10.3390/nu15040907/s1, Figure S1: The volcano plots for the differentially expressed lncRNA, circRNA and miRNA; Figure S2: The obtained significant pathways by GSVA. Table S1: The primers used for qPCR; Table S2: 48 differential pathways and their specific genes; Table S3: The specific differentially expressed genes in nine pathways.
