# Supplementary material for: Naringin Alleviates Glucose-Induced Aging by Reducing Fat Accumulation and Promoting Autophagy in Caenorhabditis elegans
Source: Nutrients. 2023 Feb 10;15(4):907. doi: 10.3390/nu15040907 (PMC9961211; doi:10.3390/nu15040907)
Supplement: Supplementary file 1 [file nutrients-15-00907-s001.zip › nutrients-2181414-supplementary.pdf]

nutrients MDPI

Supplementary Table

Table S1. The primers used for qPCR.

Gene	Primer	Product length
act-1	F: CAATGAGCTTCGTGTTGCCC<br>R: AGGGAGAGGACAGCTTGGAT	153
skn-1	F: TCAGACTCCACTGTCTCCTCT<br>R: AAACGAGTGTCTCTGTGAGTGA	139
aak-2	F: TGGCGTATTCCCAACTCCAG<br>R: GAACCACTCGTGAGCAATGAC	121
lgg-1	F: AACGCATCCAACTTCGTCCA<br>R: TCGACCTCTCCTCCATACACA	163
fat-5	F: GATCCGGTGCTGATGTTCCA<br>R: AAGGCGATGAAGGCTGACTC	116
fat-6	F: CCCACTTGTCATCTTGTGCTG<br>R: TGGCTTCCATCCGAAATAGTGAG	169
fat-7	F: CGTTGCCATCACAAGTGGAC<br>R: TCGAGTTTGCCTCCATGCTC	131
acs-2	F: AGGCTTGTAAGAGAGGAATGGC<br>R: TGACAGTTCCGAGACCCAAC	150
nhr-49	F: TCGGATTGGCAGAGGTGGAT<br>R: CCGGCTGGATTTAACGGAGAT	148
cst-1	F: ACCGTTTGCTCAAGATGGAAC<br>R: TTCGTATTCGGCTTCCGTGT	106
acdh-8	F: TCTGGAACCACCTGGAATCG<br>R: AGCATTTGGCACAAGAACGTC	126
dhs-28	F: GACCGAGCTTGATTGGGATCTT<br>R: CACTTTTGGCAGCAGCGTAG	176
ech-7	F: CCGGTATGTGAGCAAGTCAGT<br>R: TCAGCCAACAGAACAGCCTC	141
yap-1	F: GCAGGACTCTCGGAAATACCAC<br>R: GCATGGGTAACTGCTCAACATC	124
egl-44	F: TTCCGACTTGTCTGGTGACG<br>R: GCGATTAGTTCGTTCCTTCCATAC	150
unc-51	F: AGTTCGGAGGATCGGAGAGT<br>R: TGGTATGCACTTGGACCTGC	161
pmk-1	F: TACTTCATCCGACTCCACGAGA<br>R: GAGTACATTCAGCAGCACAAACAG	138
pha-4	F: AACCACGCAAGCACAGATGA<br>R: CCTGGATAACTGCTCGTTGTGT	138
acox-1.1	F: CTCGCTCGTTACCTCGTCAA<br>R: TAGTTGGCTCACTTCTGGCG	103
fasn-1	F: CTCATGGCTGACAAGTACAAGC<br>R: GCACCTCGCAATCTTCTGAC	139
acs-4	F: CCATACCAGTTGTTCAATTCGCC<br>R: GACGCACTTTCTCCAAACCTTATC	165
ech-1.2	F: AAGGCCACTTTGGATAAGCTCC<br>R: CTGTAGCAGTTCCTTCTGCCT	127

ech-6	F: TTGGCTGATGCTCTCGAAGT<br>R: GCGAACTCGTTATTGGTCATCTC	122
acox-1.4	F: TGATCTATGGAAGTGAGAAGCTCG<br>R: ATGAACGGAAAGGGTTGGGT	100
acox-1.3	F: CGCTGAGTCCTGTAGAAAGCTG<br>R: ACCACTTCTCCTGCTGCTCT	197
acox-1.2	F: CCGACATCAAGCCCTATCCAT<br>R: CTCGTAGCCAATCACTTCTCGAT	155
hlh-30	F: GAGGTGACAAACAACACCGC<br>R: GCCGCTGCTCGTCCTATAAT	144

Table S2. 48 differential pathways and their specific genes.

KEGG name	Pathway	Gene	*t*-value	
			Nar + Glu *vs.* DMSO + Glu	Glu *vs* Control
cel00010	Glycolysis / Gluconeogenesis	hxk-1,gpi-1,pfk-1.2,pfk-1.1,fbp-1,aldo-1,aldo-2,tpi-1,gpd-1,gpd-4,gpd-3,gpd-2,pgk-1,ipgm-1,enol-1,pyk-1,pyk-2,pdha-1,pdhb-1,dlat-2,dlat-1,dld-1,ldh-1,adh-5,D2063.1,sodh-2,sodh-1,alh-1,alh-4,alh-5,alh-2,alh-9,alh-12,alh-11,acs-19,C01B4.6,Y19D10A.16,R05F9.6,Y43F4B.5,C50D2.7,pck-2,pck-1	−4.83	1.03
cel00020	Citrate cycle (TCA cycle)	cts-1,acly-2,acly-1,aco-2,aco-1,idh-2,idh-1,idhb-1,idhg-1,idha-1,idhg-2,ogdh-1,dlst-1,dld-1,sucl-1,sucl-2,sucg-1,suca-1,sdha-1,sdha-2,sdhb-1,mev-1,sdhd-1,fum-1,mdh-1,mdh-2,pyc-1,pck-2,pck-1,pdha-1,pdhb-1,dlat-2,dlat-1	−20.92	−0.82
cel00030	Pentose phosphate pathway	gpi-1,gspd-1,Y57G11C.3,T25B9.9,F08F8.7,tkt-1,tald-1,rpia-1,F09E5.3,F07A11.5,R05F9.6,Y43F4B.5,R151.2,F26D11.1,C13B9.2,aldo-1,aldo-2,fbp-1,pfk-1.2,pfk-1.1	−14.05	−5.64
cel00051	Fructose and mannose metabolism	C05C8.7,ZK632.4,F52B11.2,tag-335,Y47D9A.1,gmd-2,bre-1,ger-1,K03H1.13,hxk-1,pfk-1.2,pfk-1.1,fbp-1,R04B5.5,R04B5.6,Y39G8B.1,aldo-1,aldo-2,tpi-1,W02H5.8	−4.16	−0.25
cel00052	Galactose metabolism	C01B4.6,Y19D10A.16,ZK1058.3,gale-1,rml-1,R05F9.6,Y43F4B.5,hxk-1,bgal-1,Y39G8B.1,pfk-1.2,pfk-1.1,aagr-1	−3.40	−1.55
cel00061	Fatty acid biosynthesis	pod-2,C50D2.9,fasn-1,F10G8.9,dhs-25,dhs-11,mecr-1,Y48A6B.9,acs-17,acs-15,acs-18,acs-3,acs-5,acs-2,acs-4,acs-16,acs-13	−3.30	2.32
cel00062	Fatty acid elongation	acaa-2,B0303.3,B0272.3,ech-8,ech-9,hacd-1,F54C8.1,ech-1.2,ech-1.1,ech-6,ech-7,mecr-1,Y48A6B.9,ppt-1,elo-6,elo-5,elo-3,elo-2,elo-1,elo-4,elo-9,let-767,stdh-2,stdh-1,stdh-4,stdh-3,hpo-8,art-1	−3.82	−0.41
cel00071	Fatty acid degradation	T02G5.7,kat-1,acaa-2,B0303.3,B0272.3,ech-8,ech-9,hacd-1,F54C8.1,ech-1.2,ech-1.1,ech-6,ech-7,acox-1.5,acox-1.3,acox-1.4,acox-1.6,acox-3,acox-1.1,acox-1.2,acdh-7,acdh-8,acdh-10,acdh-3,acdh-4,acdh-1,acdh-12,F54D5.7,acs-17,acs-15,acs-18,acs-3,acs-5,acs-2,acs-4,acs-16,acs-13,cpt-1,W03F9.4,cpt-2,ech-4,adh-5,D2063.1,sodh-2,sodh-1,alh-1,alh-4,alh-5,alh-2,alh-9,alh-12,alh-11	−5.33	2.43
cel00130	Ubiquinone and other terpenoid-quinone biosynthesis	tatn-1,acs-6,coq-2,coq-6,coq-3,coq-5,clk-1,hpd-1,C31H2.4	−4.48	−5.77
cel00190	Oxidative phosphorylation	ND1,ND2,ND3,ND4,ND4L,ND5,ND6,nuo-5,gas-1,nduf-2.2,nuo-2,lpd-5,nduf-5,nduf-6,nduf-7,T20H4.5,nuo-1,F53F4.10,F31D4.9,Y63D3A.7,C33A12.1,nuo-	−7.42	−6.30

		3,F45H10.3,Y54F10AM.5,nduf-9,nuo-4,F37C12.3,Y56A3A.19,C34B2.8,F44G4.2,C18E9.4,nuo-6,C25H3.9,ZK809.3,D2030.4,Y51H1A.3,C16A3.5,F59C6.5,F42G8.10,Y71H2AM.4,sdha-1,sdha-2,sdhb-1,mev-1,sdhd-1,isp-1,CYTB,cyc-1,ucr-2.2,ucr-2.3,ucr-2.1,T27E9.2,T02H6.11,F45H10.2,R07E4.3,C14B9.10,ucr-11,cox-10,COX3,COX1,COX2,cox-4,cox-5A,cox-5B,cox-6A,cox-6B,cox-6C,cox-7C,cox-11,cox-15,cox-17,cyc-2.1,cyc-2.2,atp-1,atp-2,Y69A2AR.18,F58F12.1,hpo-18,R05D3.6,ZC262.5,atp-3,ATP6,asb-2,asb-1,Y82E9BR.3,atp-5,R04F11.2,R53.4,asg-2,asg-1,vha-13,vha-12,spe-5,vha-11,vha-14,vha-8,vha-9,vha-10,vha-18,vha-15,vha-5,vha-6,unc-32,vha-7,vha-2,vha-1,vha-3,vha-4,vha-16,vha-17,vha-19,C13C4.4,pyp-1		
cel00220	Arginine biosynthesis	glna-2,glna-3,glna-1,gln-6,gln-5,gln-2,gln-3,gln-1,gdh-1,got-1.2,got-2.2,got-2.1,C32F10.8,C10C5.5,C06A6.4,C10C5.3,C10C5.4	−3.24	2.21
cel00240	Pyrimidine metabolism	pyr-1,dhod-1,umps-1,F40F8.1,C29F7.3,ndk-1,R05G6.5,apy-1,ZK563.7,mig-23,uda-1,ndx-4,ctps-1,B0001.4,F19B6.1,Y71H10B.1,upp-1,T24C12.3,Y10G11A.1,F25B5.3,cdd-1,cdd-2,rnr-1,rnr-2,F45F2.9,ZK643.2,dtmk-1,dut-1,thk-1,tyms-1,dpyd-1,dhp-2,dhp-1,upb-1	−4.38	−21.82
cel00250	Alanine, aspartate and glutamate metabolism	got-1.2,got-2.2,got-2.1,ddo-2,ddo-1,ddo-3,asns-1,asns-2,C32F10.8,agxt-1,T09B4.8,adss-1,adsl-1,unc-25,gta-1,alh-7,W07E11.1,gdh-1,alh-6,gln-6,gln-5,gln-2,gln-3,gln-1,pyr-1,glna-2,glna-3,glna-1,gfat-2,gfat-1,ppat-1	−3.90	3.72
cel00260	Glycine, serine and threonine metabolism	R102.4,mel-32,agxt-1,C13B9.2,ipgm-1,C31C9.2,F26H9.5,Y62E10A.13,T25B9.1,F08F3.4,gldc-1,gcst-1,dld-1,gcsh-2,gcsh-1,daao-1,T09B4.8,chdh-1,alh-9,Y37E3.17,C15B12.1,cbs-1,cbs-2,cth-2,cth-1,K01C8.1,Y51H7C.9,serr-1	−9.61	−3.64
cel00270	Cysteine and methionine metabolism	cysl-1,cysl-4,cysl-2,cysl-3,cth-2,cth-1,nkat-3,nkat-1,cbs-1,cbs-2,T13G4.4,metr-1,sams-1,sams-4,sams-3,sams-5,smd-1,spds-1,B0228.7,C01G10.9,ZC373.5,F58H1.3,F42F12.4,K07E1.1,T01D1.4,tatn-1,ahcy-1,Y44A6D.5,bcat-1,T09B4.8,gcs-1,E01A2.1,gss-1,cdo-1,got-1.2,got-2.2,got-2.1,mpst-1,mpst-4,mpst-2,ldh-1,mdh-1,mdh-2,C31C9.2,F26H9.5	−6.13	−5.12
cel00280	Valine, leucine and isoleucine degradation	Y44A6D.5,bcat-1,bckd-1A,bckd-1B,dbt-1,dld-1,acdh-7,acdh-8,acdh-10,ivd-1,acdh-3,acdh-4,acdh-1,acdh-9,ech-1.2,ech-1.1,ech-6,ech-7,B0272.3,ech-8,ech-9,hacd-1,F54C8.1,ard-1,acaa-2,B0303.3,pcca-1,pccb-1,mce-1,mmcm-1,hach-1,B0250.5,alh-8,alh-1,alh-4,alh-5,alh-2,alh-9,alh-12,alh-11,gad-3,gta-1,T09B4.8,mccc-1,mccc-2,ech-5,Y71G12B.10,C05C10.3,sur-5,T02G5.7,kat-1,hmgs-1	−13.83	−0.75

cel00310	Lysine degradation	aass-1,alh-9,ogdh-2,dlst-1,dld-1,F54D5.7,ech-1.2,ech-1.1,ech-6,ech-7,B0272.3,ech-8,ech-9,hacd-1,F54C8.1,T02G5.7,kat-1,C15B12.1,set-16,set-2,lin-59,met-2,mes-2,set-23,dot-1.1,dot-1.2,dot-1.4,dot-1.3,set-1,set-4,alh-1,alh-4,alh-5,alh-2,alh-12,alh-11,gbh-1,gbh-2,let-268,D2045.9	−4.33	−0.25
cel00330	Arginine and proline metabolism	odc-1,spds-1,smd-1,alh-1,alh-4,alh-5,alh-2,alh-9,alh-12,alh-11,W10C8.5,argk-1,F46H5.3,F32B5.1,ZC434.8,oatr-1,pycr-1,pycr-4,prdh-1,alh-6,alh-13,dpy-18,phy-2,got-1.2,got-2.2,got-2.1,daao-1	−11.76	2.84
cel00350	Tyrosine metabolism	got-1.2,got-2.2,got-2.1,tatn-1,hpd-1,C31H2.4,hgo-1,Y53G8B.1,gst-42,gst-43,fah-1,tyr-4,cat-2,tdc-1,basl-1,bas-1,tbh-1,adh-5,D2063.1,sodh-2,sodh-1,gad-3,fahd-1,mif-1	−7.06	−6.29
cel00360	Phenylalanine metabolism	pah-1,basl-1,bas-1,got-1.2,got-2.2,got-2.1,tatn-1,hpd-1,C31H2.4,mif-1	−15.11	−4.02
cel00380	Tryptophan metabolism	tdo-2,afmd-1,kmo-1,kynu-1,haao-1,acsd-1,alh-10,ogdh-2,dlst-1,dld-1,F54D5.7,ech-1.2,ech-1.1,ech-6,ech-7,B0272.3,ech-8,ech-9,hacd-1,F54C8.1,T02G5.7,kat-1,nkat-3,nkat-1,tph-1,basl-1,bas-1,alh-1,alh-4,alh-5,alh-2,alh-9,alh-12,alh-11,gad-3,nit-1,ctl-3,ctl-2,ctl-1	−11.35	1.02
cel00400	Phenylalanine, tyrosine and tryptophan biosynthesis	got-1.2,got-2.2,got-2.1,tatn-1,pah-1	−8.24	−2.58
cel00410	beta-Alanine metabolism	unc-25,gta-1,alh-1,alh-4,alh-5,alh-2,alh-9,alh-12,alh-11,dpyd-1,dhp-2,dhp-1,upb-1,hach-1,ech-1.2,ech-1.1,ech-6,ech-7,acox-1.5,acox-1.3,acox-1.4,acox-1.6,acox-3,acox-1.1,acox-1.2,mlcd-1,alh-8	−5.16	0.96
cel00480	Glutathione metabolism	Y7A9A.1,C53D5.5,T03D8.6,H14N18.4,C44B7.7,F22F7.7,Y38F2AR.12,gcs-1,E01A2.1,gss-1,lap-2,anp-1,gst-6,gst-5,gst-36,gst-8,gst-7,gst-38,gst-4,gst-2,gst-3,gsto-1,gsto-2,C02D5.4,gst-44,gst-9,gst-24,gst-20,gst-28,gst-39,W10C8.4,gst-1,gst-25,gst-41,gst-23,gst-10,gstk-1,gstk-2,gst-11,gst-30,gst-33,gsr-1,idh-2,idh-1,T25B9.9,gspd-1,txdc-12.1,gpx-5,gpx-3,gpx-7,gpx-2,gpx-1,prdx-6,odc-1,spds-1,rnr-1,rnr-2	−2.92	−1.31
cel00500	Starch and sucrose metabolism	aagr-1,rml-1,klo-1,gsy-1,gyg-2,gyg-1,T04A8.7,pygl-1,agl-1,C50B6.7,tre-1,tre-3,tre-5,tre-2,R05F9.6,Y43F4B.5,hxk-1,gpi-1	−4.38	−2.48
cel00510	N-Glycan biosynthesis	B0024.13,dolk-1,algn-7,algn-5,algn-13,algn-14,dpm-1,dpm-3,algn-1,algn-2,algn-11,algn-3,algn-9,algn-12,algn-6,algn-8,algn-10,stt-3,ribo-1,ostd-1,dad-1,ZK686.3,ostb-1,mogs-1,aagr-3,aagr-4,mans-3,mans-4,mans-1,mans-2,gly-13,gly-14,gly-12,aman-2,gly-20,fut-8,bre-4,gly-2	−11.84	−25.66
cel00513	Various types of N-glycan biosynthesis	algn-13,algn-14,algn-1,algn-2,algn-11,algn-3,algn-9,algn-12,stt-3,ribo-1,ostd-1,dad-1,ZK686.3,ostb-1,mans-3,mans-4,mans-1,mans-2,gly-13,gly-14,gly-12,aman-2,gly-20,fut-4,hex-1,fut-8,galt-1,hex-2,hex-3,hex-5,hex-4,fut-1,bre-4	−8.34	−8.55

cel00520	Amino sugar and nucleotide sugar metabolism	cht-1,cht-4,hex-1,W06B4.2,F59B2.3,T03F6.3,hxk-1,gfat-2,gfat-1,gna-1,F21D5.1,tag-96,C36A4.4,T05H4.4,hpo-19,Y52B11A.3,chs-2,chs-1,sqv-1,R05F9.6,Y43F4B.5,rml-1,rml-4,sqv-4,ZK1058.3,gale-1,gpi-1,C05C8.7,ZK632.4,F52B11.2,tag-335,Y47D9A.1,gmd-2,bre-1,ger-1,K03H1.13	−5.16	−5.51
cel00532	Glycosaminoglycan biosynthesis - chondroitin sulfate / dermatan sulfate	sqv-6,sqv-3,sqv-2,sqv-8,sqv-5,mig-22	−3.62	−5.17
cel00562	Inositol phosphate metabolism	vps-34,mtm-1,mtm-3,mtm-6,Y75B8A.24,pifk-1,ZC8.6,C56A3.8,sac-1,sac-2,ppk-1,ppk-3,ocrl-1,inpp-1,unc-26,age-1,daf-18,piki-1,figo-1,ppk-2,egl-8,plc-4,plc-1,plc-3,ttx-7,pisy-1,inos-1,ipp-5,lfe-2,ipmk-1,C54E4.5,alh-8,tpi-1	−2.97	−1.38
cel00592	alpha-Linolenic acid metabolism	C07E3.9,C03H5.4,ipla-3,ipla-2,acox-1.5,acox-1.3,acox-1.4,acox-1.6,acox-3,acox-1.1,acox-1.2	−7.49	1.70
cel00620	Pyruvate metabolism	acs-19,pdha-1,pdhb-1,dlat-2,dlat-1,dld-1,D2063.1,sodh-2,sodh-1,adh-5,pyk-1,pyk-2,pod-2,alh-1,alh-4,alh-5,alh-2,alh-9,alh-12,alh-11,ldh-1,F32D8.12,Y17G7B.3,men-1,pyc-1,mdh-1,mdh-2,fum-1,pck-2,pck-1,icl-1,T02G5.7,kat-1	−4.85	1.78
cel00630	Glyoxylate and dicarboxylate metabolism	icl-1,acs-19,mdh-1,mdh-2,cts-1,aco-2,aco-1,T02G5.7,kat-1,mce-1,pcca-1,pccb-1,mmcm-1,F41E6.5,ctl-3,ctl-2,ctl-1,pgph-1,pgph-2,pgph-3,C45E5.1,agxt-1,gln-6,gln-5,gln-2,gln-3,gln-1,mel-32,gldc-1,gcst-1,dld-1,gcsh-2,gcsh-1,C05D11.5,C13B9.2,afmd-1	−10.12	5.09
cel00640	Propanoate metabolism	acs-19,bckd-1A,bckd-1B,dbt-1,dld-1,acox-1.5,acox-1.3,acox-1.4,acox-1.6,acox-3,acox-1.1,acox-1.2,ech-1.2,ech-1.1,ech-6,ech-7,hach-1,pod-2,mlcd-1,gta-1,pcca-1,pccb-1,C32E8.9,mce-1,mmcm-1,sucl-1,sucl-2,sucg-1,suca-1,alh-8,ldh-1	−21.99	0.38
cel00650	Butanoate metabolism	T02G5.7,kat-1,B0272.3,ech-8,ech-9,hacd-1,F54C8.1,ech-1.2,ech-1.1,ech-6,ech-7,Y45G12B.3,unc-25,gta-1,alh-7,hmgs-1,Y71G12B.10,C05C10.3,sur-5	−6.24	0.55
cel00983	Drug metabolism - other enzymes	hprt-1,T22D1.3,gmps-1,xdh-1,hap-1,ges-1,cest-35.2,cest-33,ugt-46,ugt-55,ugt-56,ugt-58,ugt-48,ugt-50,ugt-61,ugt-62,ugt-23,ugt-6,ugt-18,ugt-15,ugt-59,Y105E8B.9,cdd-1,cdd-2,dpyd-1,dhp-2,dhp-1,upb-1,upp-1,B0001.4,F19B6.1,thk-1,umps-1,F40F8.1,C29F7.3,rnr-1,rnr-2,ndk-1,R05G6.5,dut-1,gst-6,gst-5,gst-36,gst-8,gst-7,gst-38,gst-4,gst-2,gst-3,gsto-1,gsto-2,C02D5.4,gst-44,gst-9,gst-24,gst-20,gst-28,gst-39,W10C8.4,gst-1,gst-25,gst-41,gst-23,gst-10	−4.40	−1.60
cel01040	Biosynthesis of unsaturated fatty acids	elo-6,elo-5,elo-3,elo-2,elo-1,elo-4,elo-9,let-767,stdh-2,stdh-1,stdh-4,stdh-3,hpo-8,art-1,fat-7,fat-6,fat-5,acox-1.5,acox-1.3,acox-1.4,acox-1.6,acox-3,acox-1.1,acox-1.2,dhs-28,maoc-1,daf-22	−3.94	1.46

cel02010	ABC transporters	abt-2,abt-4,haf-6,haf-9,haf-7,haf-4,haf-2,haf-3,pgp-2,pgp-9,pgp-13,pgp-11,pgp-12,pgp-14,pgp-3,pgp-4,pgp-1,pgp-6,pgp-5,hmt-1,abtm-1,mrp-2,mrp-1,cft-1,mrp-5,pmp-4,pmp-2,pmp-1,pmp-3,pmp-5	−3.59	−0.22
cel03050	Proteasome	rpn-3,psmd-9,rpn-5,rpn-6.1,rpn-6.2,rpn-7,rpn-8,rpn-9,rpn-11,F37A4.5,rpn-12,dss-1,rpn-10,rpn-1,rpn-2,rpn-13,rpt-1,C10G11.8,rpt-2,rpt-6,F56F11.4,rpt-4,rpt-5,rpt-3,Y66D12A.9,C14C10.5,T28B8.3,T28B8.4,pas-1,pas-2,pas-3,pas-4,pas-5,pas-6,pas-7,pbs-1,pbs-2,pbs-3,pbs-4,pbs-5,pbs-6,pbs-7	−2.78	−3.51
cel04020	Calcium signaling pathway	ncx-1,ncx-2,mca-3,mca-2,mca-1,gar-3,gar-1,gar-2,dop-1,ser-7,gsa-1,acy-1,kin-1,sca-1,stim-1,orai-1,egl-19,cca-1,nmr-1,unc-68,csq-1,K09A9.6,mgl-2,ser-1,tkr-2,trhr-1,ckr-2,ckr-1,egl-30,let-756,let-23,ver-3,ver-1,svh-2,plc-4,egl-8,plc-3,plc-1,itr-1,cup-5,sphk-1,mcu-1,vdac-1,ant-1.3,ant-1.4,F25B4.7,ant-1.2,ant-1.1,C47E12.2,R07E3.4,T22F3.12,cmd-1,cal-2,cal-4,cal-3,cal-1,K03A1.4,cal-7,B0563.7,Y50D7A.3,C14B9.8,Y67D8A.2,mlck-1,cmk-1,unc-43,tax-6,cnb-1,pde-1,lfe-2,pkc-2	3.50	4.02
cel04141	Protein processing in endoplasmic reticulum	sec-61.A,sec-61.B,sec-61.G,C18E9.2,dnj-29,ribo-1,ostd-1,dad-1,ZK686.3,ostb-1,stt-3,mogs-1,T24H7.2,T14G8.3,hsp-3,hsp-4,dnj-20,F54F2.9,dnj-7,dnj-28,dnj-27,enpl-1,aagr-3,aagr-4,ZK1307.8,cnx-1,pdi-3,crt-1,mans-4,mans-1,mans-2,mans-3,ile-2,ile-1,sec-12,sar-1,npp-20,sec-23,sec-24.2,sec-24.1,uggt-1,C47E12.3,F10C2.5,ZC506.1,pdi-2,pdi-1,C14B9.2,pdi-6,Y49E10.4,ero-1,Y105E8A.2,trap-1,trap-2,trap-3,trap-4,Y54G2A.18,tram-1,cup-2,der-2,ubxn-1,ubxn-4,ubxn-6,ubxn-2,cdc-48.2,cdc-48.1,npl-4.2,npl-4.1,ufd-1,hsp-1,F44E5.4,hsp-70,F44E5.5,F11F1.1,dnj-12,dnj-19,dnj-1,dnj-14,hsp-90,hsp-110,bag-1,unc-23,ufd-3,rad-23,ubql-1,png-1,atx-3,ufd-2,pek-1,gcn-2,eif-2alpha,skn-1,atf-4,atf-6,Y56A3A.2,xbp-1,ire-1,nsy-1,mek-1,jnk-1,kgb-1,kgb-2,marc-6,ubc-6,ubc-26,ubc-15,ubc-7,ubc-14,sel-11,rnf-5,sel-1,hrdl-1,chn-1,let-70,pdr-1,rbx-1,cul-1,cul-6,skr-1,skr-21,skr-5,skr-4,skr-3,skr-2,skr-20,skr-6,skr-12,skr-9,skr-13,skr-8,skr-15,skr-10,skr-7,skr-14,skr-17,skr-16,skr-18,skr-19	−5.43	−5.80
cel04145	Phagosome	act-4,act-3,act-1,act-2,act-5,syx-18,sec-22,rab-5,eea-1,vps-34,hgrs-1,vha-13,vha-12,spe-5,vha-11,vha-14,vha-8,vha-9,vha-10,vha-17,vha-5,vha-6,unc-32,vha-7,vha-16,vha-18,vha-15,vha-2,vha-1,vha-3,vha-4,vha-19,rab-7,dhc-1,che-3,dyci-1,dli-1,mec-12,tba-4,tba-1,tba-5,tba-6,tba-2,tba-7,tba-9,tba-8,tbb-2,ben-1,tbb-1,tbb-6,mec-7,tbb-4,lmp-1,cpl-1,sec-61.A,sec-61.B,sec-61.G,crt-1,cnx-1,pat-3,ced-10,mig-2,rac-2	−3.60	−3.86
cel04146	Peroxisome	prx-3,prx-19,pmp-2,pmp-1,prx-5,prx-14,prx-13,prx-12,prx-10,prx-2,T18D3.9,ZK470.1,pxmp-4,prx-11,hacl-1,C24A3.4,ZK892.4,ZK550.6,ZK550.5,acox-1.5,acox-1.3,acox-1.4,acox-1.6,acox-3,acox-1.1,acox-1.2,dhs-28,maoc-1,daf-	−4.37	23.90

		22,Y25C1A.13,F58A6.1,pmp-4,pmp-3,pmp-5,acs-17,acs-15,acs-18,acs-3,acs-5,acs-2,acs-4,acs-16,acs-13,ech-4,ndx-9,F25E2.3,B0395.3,T20B3.1,F41E7.6,mlcd-1,acl-7,ads-1,fard-1,mvk-1,F32D8.13,agxt-1,daao-1,ddo-2,ddo-1,ddo-3,idh-2,idh-1,C15B12.1,Y71G12B.10,F41E6.5,ctl-3,ctl-2,ctl-1,sod-1,sod-5,sod-4,sod-2,sod-3,prdx-2,gstk-1,gstk-2,xdh-1,dhs-13,dhrs-4		
cel04212	Longevity regulating pathway - worm	jkk-1,jnk-1,kgb-1,kgb-2,ins-7,daf-2,ist-1,age-1,daf-18,akt-2,akt-1,daf-16,sir-2.1,hcf-1,smk-1,mtl-1,sod-2,sod-3,ctl-3,ctl-2,ctl-1,gst-6,gst-5,gst-36,gst-8,gst-7,gst-38,gst-4,gst-2,gst-3,gsto-1,gsto-2,C02D5.4,gst-44,gst-9,gst-24,gst-20,gst-28,gst-39,W10C8.4,hsb-1,ddl-1,ddl-2,hsf-1,hsp-16.1,hsp-16.11,hsp-16.49,hsp-16.48,hsp-12.6,sip-1,sek-1,pmk-2,pmk-1,pmk-3,skn-1,gcs-1,let-363,R04A9.7,rsks-1,pha-4,unc-51,lgg-1,ftt-2,par-5,F49C12.12,C46G7.1,tcer-1,kri-1,lipl-4,lips-17,daf-36,daf-9,daf-12,fard-1,nhr-80,fat-7,fat-6,fat-5,vhl-1,hif-1,parp-1,parp-2,clpp-1,haf-1,dve-1,ubl-5,atf-4,timm-23,hsp-6,hsp-60	3.03	3.57
cel04310	Wnt signaling pathway	mom-1,mom-2,cwn-1,egl-20,sfrp-1,mom-5,mig-1,cfz-2,lin-17,dsh-1,dsh-2,kin-3,kin-10,gsk-3,C44H4.6,R03D7.5,bar-1,wrm-1,hmp-2,pry-1,kin-19,pop-1,ctbp-1,unc-37,jac-1,cbp-1,ruvb-1,sma-4,daf-3,Y105C5A.24,mom-4,lit-1,cyd-1,sel-12,hop-1,spe-4,kin-1,siah-1,skr-1,skr-21,skr-5,skr-4,skr-3,skr-2,skr-20,skr-6,skr-12,skr-9,skr-13,skr-8,skr-15,skr-10,skr-7,skr-14,skr-17,skr-16,skr-18,skr-19,lin-23,cul-1,cul-6,rbx-1,lin-18,vang-1,prkl-1,daam-1,rho-1,ced-10,mig-2,rac-2,jnk-1,kgb-1,kgb-2,egl-8,unc-43,tax-6,cnb-1,pkc-2	3.67	0.18
cel04350	TGF-beta signaling pathway	F35B12.10,dbl-1,sma-6,drag-1,daf-4,sma-2,sma-3,sma-4,daf-3,lin-35,efl-1,dpl-1,cbp-1,rbx-1,cul-1,cul-6,skr-1,skr-21,skr-5,skr-4,skr-3,skr-2,skr-20,skr-6,skr-12,skr-9,skr-13,skr-8,skr-15,skr-10,skr-7,skr-14,skr-17,skr-16,skr-18,skr-19,mpk-1,rho-1,let-502,paa-1,let-92,R04A9.7,rsks-1	3.86	-3.32
cel04392	Hippo signaling pathway - multiple species	cdh-1,nfm-1,frm-7,cst-1,cst-2,sav-1,wts-1,mob-1,yap-1,egl-44,mob-2,pak-1,max-2	8.39	1.70
cel04512	ECM-receptor interaction	let-2,lam-3,epi-1,lam-1,lam-2,agr-1,pat-3,dgn-1	3.27	0.96

Table S3. The specific differentially expressed genes in nine pathways.

Pathway	Up-regulated gene	Down-regulated gene
Fatty acid biosynthesis	acs-2,acs-3	dhs-25,fasn-1,Y48A6B.9,acs-4
Fatty acid elongation		art-1,ech-6,ech-7,elo-1,elo-2,elo-6,let-767,B0272.3,Y48A6B.9,ech-1.2
Fatty acid degradation	acs-2,sodh-1,D2063.1,acs-3,W03F9.4	alh-4,alh-9,alh-12,ech-4,ech-6,ech-7,kat-1,B0272.3,acox-1.1,acox-1.2,acox-1.3,acox-1.4,F54D5.7,cpt-2,acs-4,acdh-3,T02G5.7,ech-1.2
alpha-Linolenic acid metabolism		acox-1.1,acox-1.2,acox-1.3,acox-1.4,ipla-3
Biosynthesis of unsaturated fatty acids		art-1,dhs-28,elo-1,elo-2,elo-6,fat-5,fat-7,let-767,acox-1.1,acox-1.2,acox-1.3,acox-1.4
Longevity regulating pathway	gst-3,ist-1,jkk-1,lgg-1,pha-4,pmk-1,pmk-2,skn-1,unc-51	ctl-2,fat-5,fat-7,gst-7,hsp-60,gsto-1,lips-17
Wnt signaling pathway	cfz-2,cwn-1,egl-20,jac-1,lin-17,skr-12,skr-13,daam-1,siah-1,prkl-1	dsh-2
TGF-beta signaling pathway	daf-4,skr-12,skr-13,drag-1	
Hippo signaling pathway	egl-44,frm-7,nfm-1,yap-1,cst-1	

Supplementary Figures

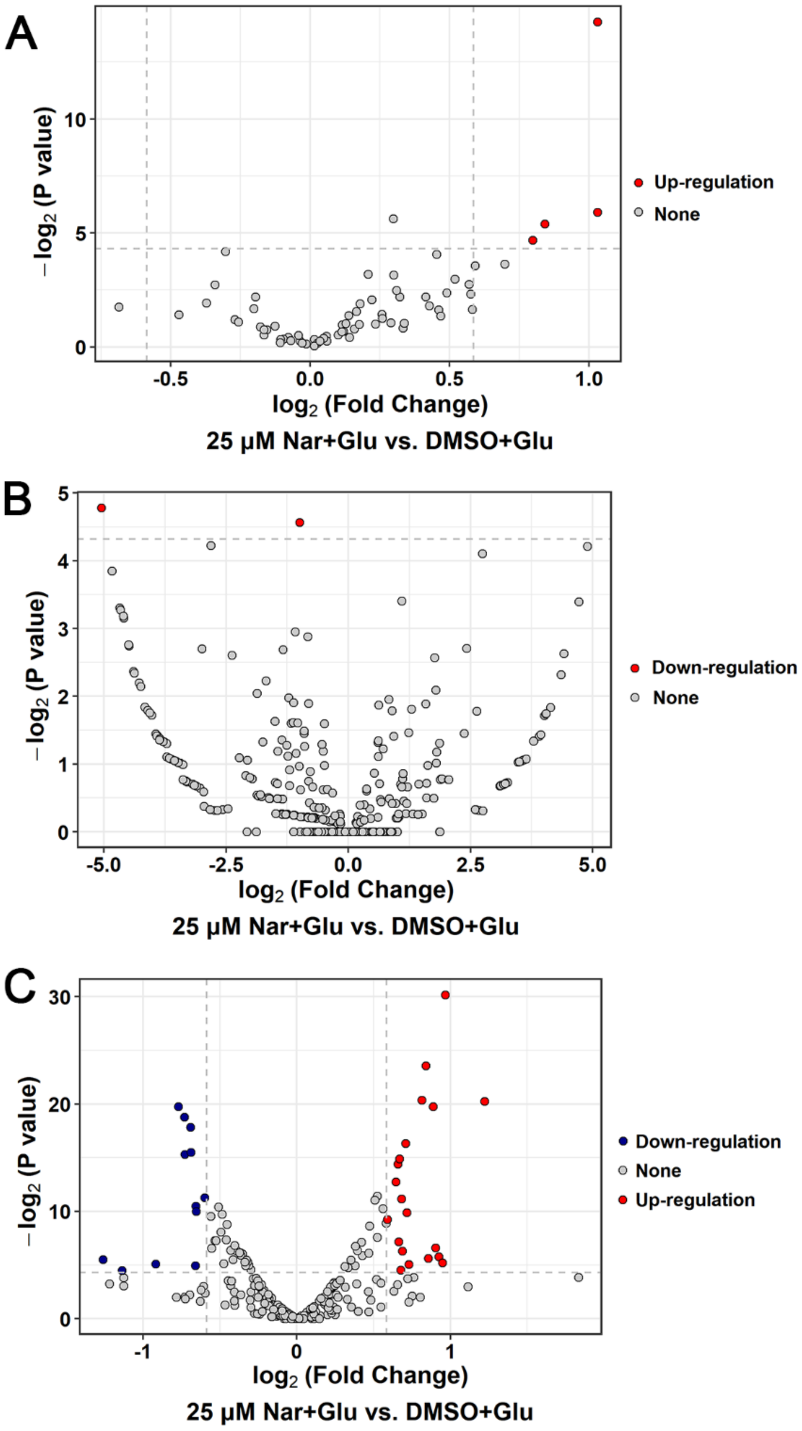

Figure S1. The volcano plots for **(A)** the differentially expressed up-regulated lncRNAs, **(B)** down-regulated circRNAs and **(C)** the up- and down-regulated miRNAs.

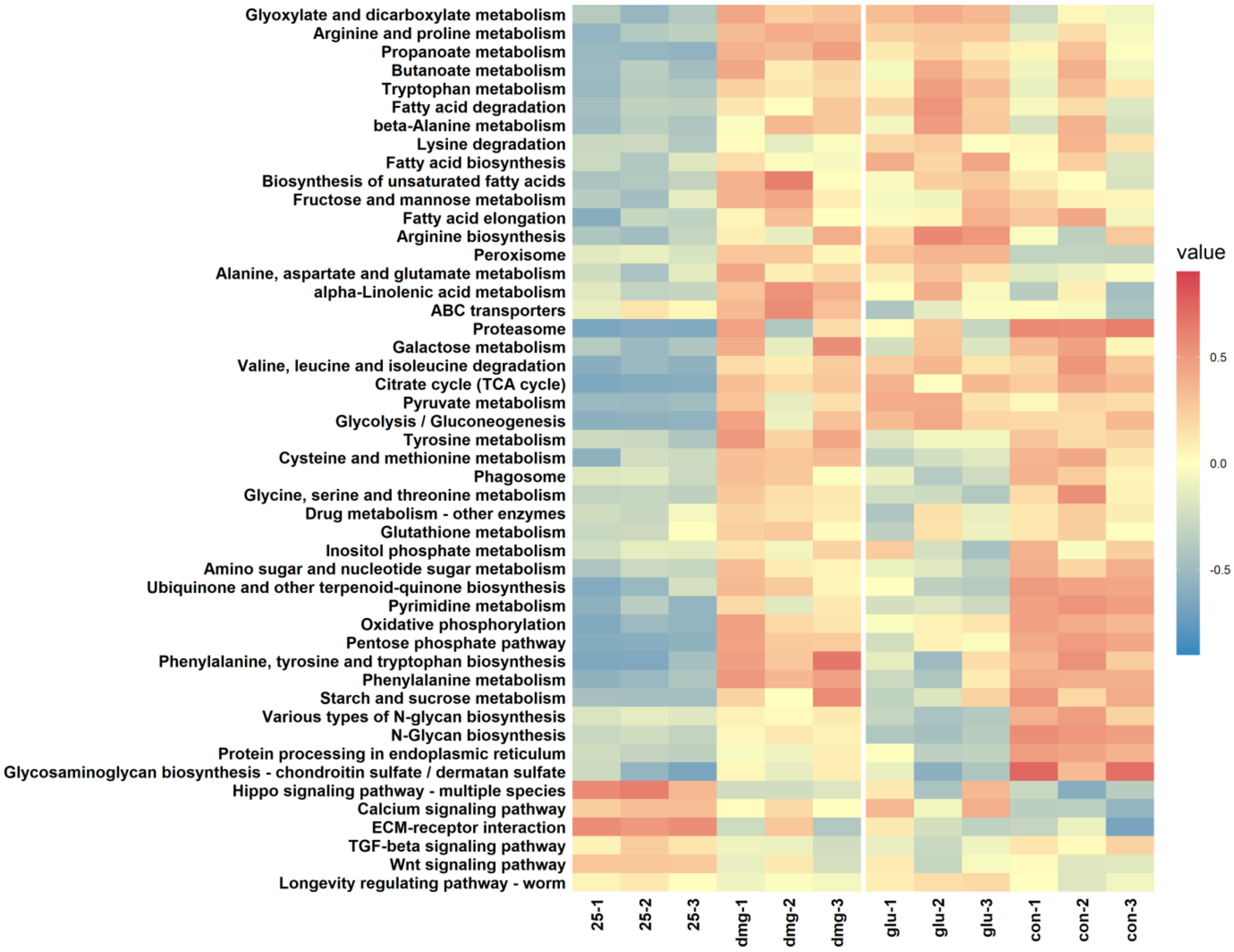

Figure S2. The obtained significant pathways by GSVA.
